# Insights from metagenomics into gut microbiome associated with acute coronary syndrome therapy

**DOI:** 10.3389/fmicb.2024.1369478

**Published:** 2024-07-05

**Authors:** Yuee Guan, Shuru Zhao, Jing Li, Wenqian Zhang, Zhonghao Guo, Yi Luo, Xiaofei Jiang, Jun Li, Jianxiong Liu, Xi Chen, Zicheng Zhao, Zhe Zhang

**Affiliations:** ^1^Department of Cardiology, Zhuhai People’s Hospital (Zhuhai Clinical Medical College of Jinan University), Zhuhai, China; ^2^Shenzhen Byoryn Technology Co., Ltd., Shenzhen, China; ^3^University of Science and Technology of China, Hefei, China; ^4^Department of Cardiology, The Second Affiliated Hospital, Xi’an Jiaotong University, Xi'an, China; ^5^Department of Computer Science, City University of Hong Kong, Kowloon Tong, China; ^6^School of Medicine, Xi’an Jiaotong University, Xi’an, China; ^7^Department of Cardiology, The First Affiliated Hospital of Jinan University, Guangzhou, China; ^8^Department of Cardiology, The Zhuhai National Hi-tech Industrial Development District People’s Hospital (Zhuhai People’s Hospital Medical Group, High-tech Zone), Zhuhai, China

**Keywords:** acute coronary syndrome, gut microbiota, metagenome, intestinal, metabolism, statin

## Abstract

Acute coronary syndrome (ACS) is a predominant cause of mortality, and the prompt and precise identification of this condition is crucial to minimize its impact. Recent research indicates that gut microbiota is associated with the onset, progression, and treatment of ACS. To investigate its role, we sequenced the gut microbiota of 38 ACS patients before and after percutaneous coronary intervention and statin therapy at three time points, examining differential species and metabolic pathways. We observed a decrease in the abundance of *Parabacteroides*, *Escherichia*, and *Blautia* in patients after treatment and an increase in the abundance of *Gemalla*, *Klebsiella variicola*, *Klebsiella pneumoniae*, and others. Two pathways related to sugar degradation were more abundant in patients before treatment, possibly correlated with disorders of sugar metabolism and risk factors, such as hyperglycemia, insulin resistance, and insufficient insulin secretion. Additionally, seven pathways related to the biosynthesis of vitamin K2 and its homolog were reduced after treatment, suggesting that ACS patients may gradually recover after therapy. The gut microbiota of patients treated with different statins exhibited notable differences after treatment. Rosuvastatin appeared to promote the growth of anti-inflammatory bacteria while reducing pro-inflammatory bacteria, whereas atorvastatin may have mixed effects on pro-inflammatory and anti-inflammatory bacteria while increasing the abundance of *Bacteroides*. Our research will provide valuable insights and enhance comprehension of ACS, leading to better patient diagnosis and therapy.

## Introduction

Acute Coronary Syndrome, a subcategory of coronary artery disease, is characterized by frequently presented syndromes, including angina, myocardial infarction, or sudden cardiac death (Sanchis-Gomar et al., 2016; Kimura et al., 2019; Ralapanawa and Sivakanesan, 2021). With global modernization, the prevalence of ACS has reached a pandemic level (Ruff and Braunwald, 2011). According to the Report on Cardiovascular Health and Diseases in China (2021), the mortality rate of coronary artery disease and acute myocardial infarction has almost doubled in the past decade, with the total prevalence continuously growing nationwide (Hu, 2022). It is also the third leading cause of mortality worldwide and is associated with 17.8 million deaths every year (Brown et al., 2020), constituting an increasing public health burden. Common approaches for treating ACS include surgery, antiplatelet and antianginal medications, as well as risk factor management (Braun et al., 2018). The prognosis of ACS is considered dynamic and complicated to determine, as it is associated with heart function, blood biomarkers level, risk factors, etc. (Xu and Yang, 2020).

Recent studies have highlighted the involvement of gut microbiome in ACS development. Some evidence suggests that trimethylamine N-oxide (TMAO) in serum, a metabolite produced by gut microbiome from certain dietary nutrients, is linked to ACS onset and coronary atherosclerotic plaque burden (Li et al., 2017, 2019a; Gao et al., 2020). ACS can also be accompanied by specific alterations in gut microbial composition, notably the increasing ratio of *Firmicutes/Bacteroidetes* and the increasing abundance of *Firmicutes*, *Proteobacteria*, *Gammaproteobacteria*, and *Aerococcaceae* (Alhmoud et al., 2019; Gao et al., 2020; Tascanov et al., 2020; Sawicka-Smiarowska et al., 2021). Another study shows that patients with symptomatic atherosclerosis had an increase in the abundance of genus *Collinsella*, while controls had an increase in *Eubacterium* and *Roseburia* (Ramírez-Macías et al., 2022). In addition, by modulating gut microbiome composition and metabolism, the Mediterranean diet has a potential effect in primary and secondary prevention of ACS because it contains more antioxidants, nitrate, and fibre as well as less saturated/trans fatty acids, sodium, and phosphate (Foscolou et al., 2019; Fernández et al., 2021; Kouvari et al., 2022; Ramírez-Macías et al., 2022). However, most previous studies used 16S rRNA amplicon sequencing to profile the bacterial community, which can result in limited taxonomic resolution and unreliable functional potential inferences (Heidrich and Beule, 2022). Moreover, there is a lack of studies investigating how the gut microbiome alters after common ACS treatments.

Shotgun metagenomic sequencing allows researchers to sequence thousands of organisms in parallel and detect very low abundance members of the microbial community (Durazzi et al., 2021). To address the gap mentioned above, we conducted metagenomic sequencing on the gut microbiome of 38 patients with acute coronary syndrome and an equal number of healthy controls who were family members. We analyzed the possible association between microbial composition and ACS prognosis stages before treatment, one and two months after treatment. We observed community-wide differences in metagenomic composition as treatment progressed and identified species-specific and functional pathways associations with these differences.

## Results

### ACS cohort characteristics and quality control of sequencing data

In this study, we enrolled 38 patients (age: 54.55 ± 0.9561 years) diagnosed with various types of ACS, including 8 with unstable angina, 19 with ST-elevation myocardial infarctions (STEMI), 9 with non-ST-elevation myocardial infarction (NSTEMI) and 2 without clear classification (Supplementary Table S1). Patients with left ventricular systolic dysfunction (ejection fraction <50%) were excluded (Table 1). We collected clinical information on the patients’ serum lipid profile and serum myocardial enzyme spectrum. We detected the cTnT index in 35 patients, with 23 patients having a result higher than 0.5, which indicates myocardial infarction (Supplementary Table S1). The levels of triglycerides, cholesterol (Chol; *p* < 0.0001), high-density lipoprotein (HDL; *p* < 0.0001), and low-density lipoprotein (LDL) in patients one month after treatment (ACS-post1) and two months after treatment (ACS-post2) were lower than before treatment (ACS-pre) (Table 1).

**Table 1:** Clinical indicators at various time points during treatment.

Clinical indicators	ACS patients (*n* = 38)			*p*-value
	ACS-pre	ACS-post1	ACS-post2	
TG (0–1.7 mmol/L)	1.68 ± 0.91	1.37 ± 0.89	1.37 ± 0.65	0.2169
Chol (0–5.18 mmol/L)	5.09 ± 1.08	3.85 ± 1.18	3.60 ± 1.03	6.341 × 10^−5^
HDL (1.29–1.55 mmol/L)	1.09 ± 0.26	1.08 ± 0.26	1.08 ± 0.26	0.991
LDL (0–3.37 mmol/L)	3.32 ± 0.87	2.17 ± 0.80	1.90 ± 0.80	3.575 × 10^−6^

Values are presented as mean ± SD. All *p*-values are from the *t*-test.

M, male; F, female; TG, triglycerides; Chol, cholesterol; HDL, high-density lipoprotein; LDL, low-density lipoprotein; ALB, albumin; Cr, Creatinine; cTnT, cardiac troponin T; BNP, B-type natriuretic peptides; EF, ejection fraction; HbA1c, hemoglobin A1c.

The normal value range of the indicator is in brackets (*p < 0.05, **p < 0.01, and ***p < 0.001).

We collected a total of 147 stool samples, including 38 from ACS-pre, 38 from ACS-post1, 33 from ACS-post2, and 38 from healthy controls who were family members of patients with evenly distributed ages (50.05 ± 1.64 years; Supplementary Table S1). The healthy group consisted of the patients’ partners, ensuring that both cohorts shared similar living areas and diets. After performing quality control and host sequence decontamination, we retained 139 samples, filtering out 1 from control and 7 from patients (1 from ACS-pre, 5 from ACS-post1, and 1 from ACS-post2). After filtering, the mean number of reads per sample was 77.53 million pairs (Supplementary Figure S1).

### Alterations in gut microbiota among the four groups

**Figure 1:**
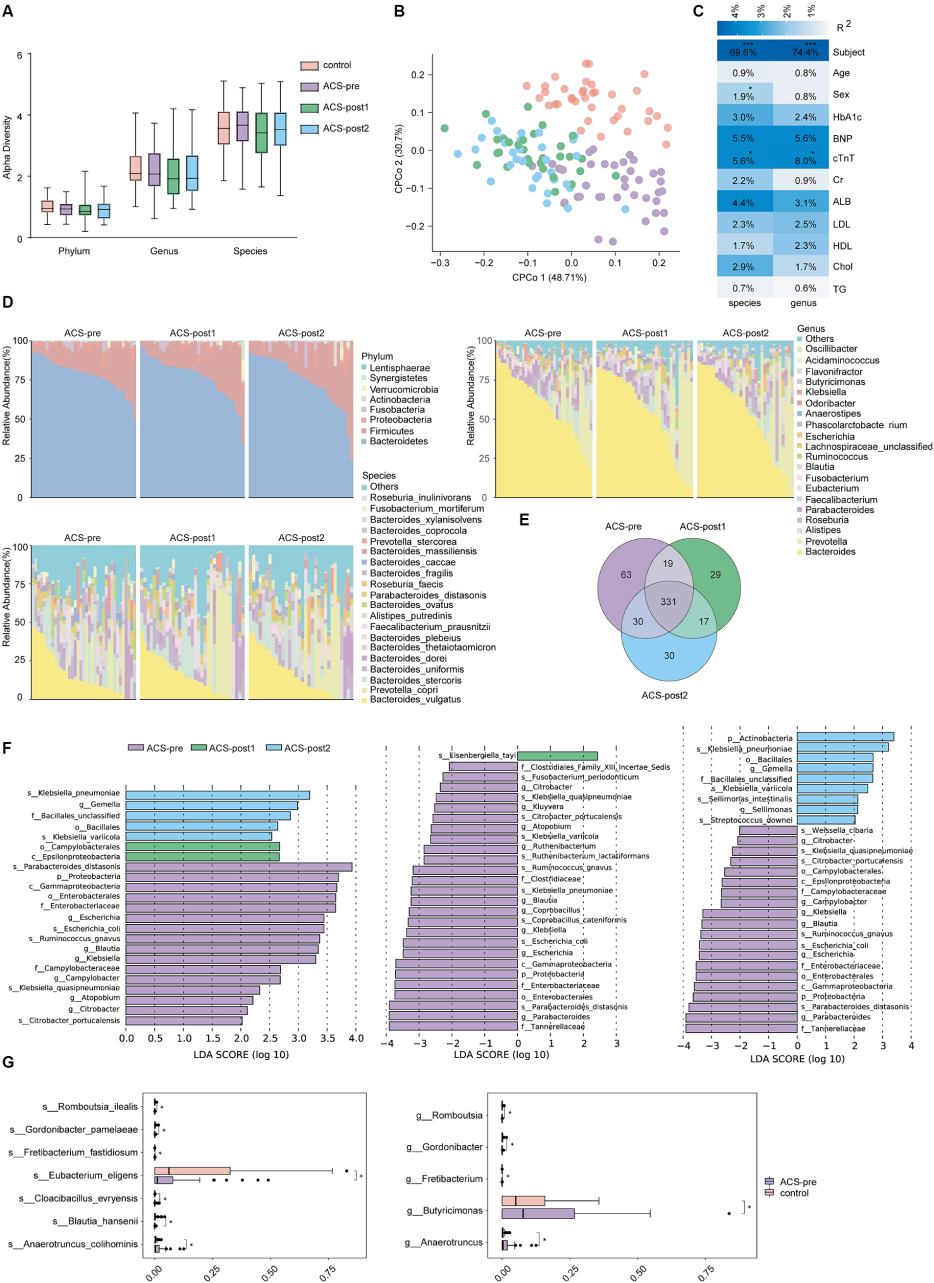
Alterations in gut microbiota among the four groups. **(A)** α-diversity (Shannon index) among the four groups was analyzed at the phylum, genus and species levels. **(B)** Constrained Principal Coordinates Analysis (CPCoA) of Euclidean distance on all samples based on species level. PERMANOVA test was used to detect the independent effects of clinical features on microbial community (Euclidean distance). **(C)** Correlation of clinical indicators and gut microbiota. R-square statistics were presented on each cell. Clinical features included subject, age, sex, TG, triglycerides; Chol, cholesterol; HDL, high-density lipoprotein; LDL, low-density lipoprotein; ALB, albumin; Cr, Creatinine; cTnT, cardiac troponin T; BNP, B-type natriuretic peptides; HbA1c, hemoglobin A1c. **(D)** Relative abundance of gut microbiota on phylum, genus, and species levels in ACS patients at three time points. **(E)** Venn diagram on species level in individuals from various stages. **(F)** Biomarkers of ACS patients at different stages of treatment (LDA score threshold >2). **(G)** Differential species between healthy controls and ACS patients before treatment (*adjust *p* < 0.05, **adjust *p* < 0.01, and ***adjust *p* < 0.001, *t*-test, Benjamini–Hochberg FDR).

We calculated α-diversity (Shannon index) and found no significant difference between the different groups (Figure 1A). We conducted Constrained Principal Coordinates Analysis (CPCoA) based on Euclidean distance and observed obvious separation between before and after treatment, as well as between controls and patients (Figure 1B). The cTnT index explained 5.6 and 8.0% of the variation in the relative abundance at species and genus levels, respectively (*p* < 0.05; Figure 1C).

To identify differences in gut microbiota between patients before and after treatment, we analyzed the microbiota composition and found that the dominant gut microbiota and their abundances were similar across the three stages of treatment (Figure 1D). The dominant phyla were *Bacteroidetes*, *Firmicutes*, and *Proteobacteria*, with the *Firmicutes*/*Bacteroidetes* ratio increasing after treatment (ACS-pre: 0.19, ACS-post1: 0.23, ACS-post2: 0.22; Supplementary Table S4). The most common bacterial genera were *Bacteroides*, *Prevotella*, and *Alistipes*, and the most common bacterial species were *Bacteroides vulgatus*, *Prevotella copri*, and *Bacteroides stercoris* in the gut of ACS patients before and after treatment (Figure 1D). The shared microbial species among patients in different stages of treatment accounted for 63.78% of the proportion, with 71.57, 73.67, and 76.32% of gut microbial species shared between ACS-pre and ACS-post1, ACS-pre and ACS-post2, and ACS-post1 and ACS-post2, respectively (Figure 1E).

When identifying biomarkers with the potential to distinguish therapy phases using LEFSe, we obtained 23 differential bacteria, including six genera and five species that were significantly more abundant in ACS-pre: *Escherichia* (*p* = 0.004), *Blautia* (*p* = 0.006), *Klebsiella* (*p* = 0.013), *Campylobacter* (*p* = 0.015), *Atopobium* (*p* = 0.027), *Citrobacter* (*p* = 0.021), *Klebsiella quasipneumoniae* (*p* = 0.003), *Escherichia coli* (*p* = 0.003), *Citrobacter portucalensis* (*p* = 0.011), *Ruminococcus gnavus* (*p* = 0.014), and *Parabacteroides distasonis* (*p* = 0.028). The genus *Gemalla* (*p* = 0.018), species *Klebsiella variicola* (*p* = 0.007), and *Klebsiella pneumoniae* (*p* = 0.018) were significantly more abundant in ACS-post2, while the class *Epsilonproteobacteria* (*p* = 0.016) was significantly more abundant in ACS-post1 (Figure 1F; Supplementary Table S5). Furthermore, we compared the three groups pairwise, revealing additional significant bacteria differences. The relative abundances of *Parabacteroides* (*p* = 0.036), *Kluyvera* (*p* = 0.030), *Ruthenibacterium* (*p* = 0.044), *Coprobacillus* (*p* = 0.041), *Proteobacteria* (*p* = 0.034), and *Gammaproteobacteria* (*p* = 0.004) were significantly lower one month after treatment than before treatment. The relative abundances of *Sellimonas* (*p* = 0.047) and *Actinobacteria* (*p* = 0.046) were significantly higher 2 months after treatment than before treatment (Figure 1F; Supplementary Table S5). Based on the results of species diversity and differential species, it can be inferred that there was little change in the composition of gut microbiota between patients after one month of treatment and after two months of treatment.

The comparison between controls and patients revealed significant species changes that could be driven by ACS. We observed a significant increase in the relative abundance of *Anaerotruncus colihominis* (*p* = 0.0163), *Cloacibacillus evryensis* (*p* = 0.021), and *Fretibacterium fastidiosum* (*p* = 0.033), while a decrease in *Gordonibacter pamelaeae* (*p* = 0.037), *Eubacterium eligens* (*p* = 0.027), *Blautia hansenii* (*p* = 0.032), and *Romboutsia ilealis* (*p* = 0.025) in ACS-pre patients (Figure 1G; Supplementary Table S5).

### Gut microbial functions vary with treatment phases

**Figure 2:**
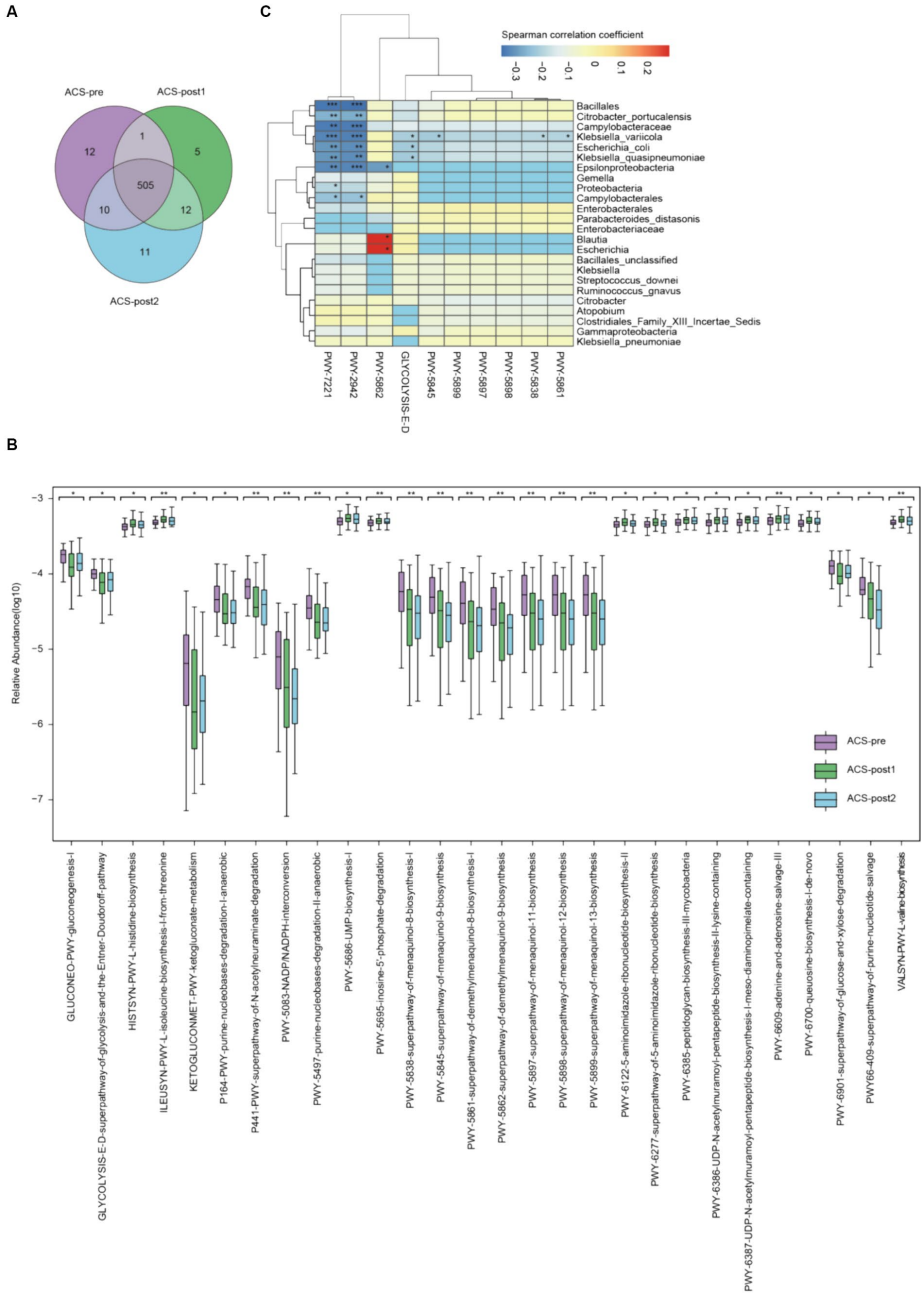
Alterations of functional pathways in ACS patients before and after treatment. **(A)** Venn diagram of metabolic pathways in ACS samples from various stages. **(B)** Significantly differential pathways of ACS patients before and after treatment (*adjust *p* < 0.05, **adjust *p* < 0.01, and ***adjust *p* < 0.001, Kruskal–Wallis test, Benjamini–Hochberg FDR). **(C)** Association between microbiome composition and metabolic pathways. Correlation values (Spearman) were presented on each cell by various colors and significance also emerged (*p < 0.05, **p < 0.01, and ***p < 0.001, *t*-test).

The gut microbiota of ACS patients participated in 556 metabolic pathways. The richness of functional pathways in ACS patients decreased after treatment but gradually restored thereafter (Figure 2A). The top abundant pathways (Supplementary Figure S2) included dTDP-β-L-rhamnose biosynthesis (DTDPRHAMSYN-PWY), adenosine ribonucleotides biosynthesis (PWY-7219), conversion from glucose to pyruvate (PWY-1042), uridine monophosphate (UMP) biosynthesis (PWY-5686), adenine and adenosine salvage III (PWY-6609).

We identified 28 pathways with statistically significant differences (*p* < 0.05; Figure 2B; Supplementary Table S9). Amino acid metabolisms, such as valine (VALSYN-PWY), histidine (HISTSYN-PWY), and isoleucine (ILEUSYN-PWY) biosynthesis, had significantly increased relative abundances in ACS patients after treatment. Two pathways related to sugar degradation were more statistically abundant in patients before than after treatment, including glucose (GLYCOLYSIS-E-D) and xylitol degradation (PWY-6901). Pathways related to sugar molecule biosynthesis were impaired after treatment, namely GLUCONEO-PWY and PWY-6385. Seven pathways reduced after treatment were separately involved in the biosynthesis of menaquinol-8, menaquinol-9, menaquinol-11, menaquinol-12, menaquinol-13, demethylmenaquinol-8, and demethylmenaquinol-9, which are important for the biosynthesis of vitamin K2 and its homolog.

### Alterations in gut microbiota affected by drugs

**Figure 3:**
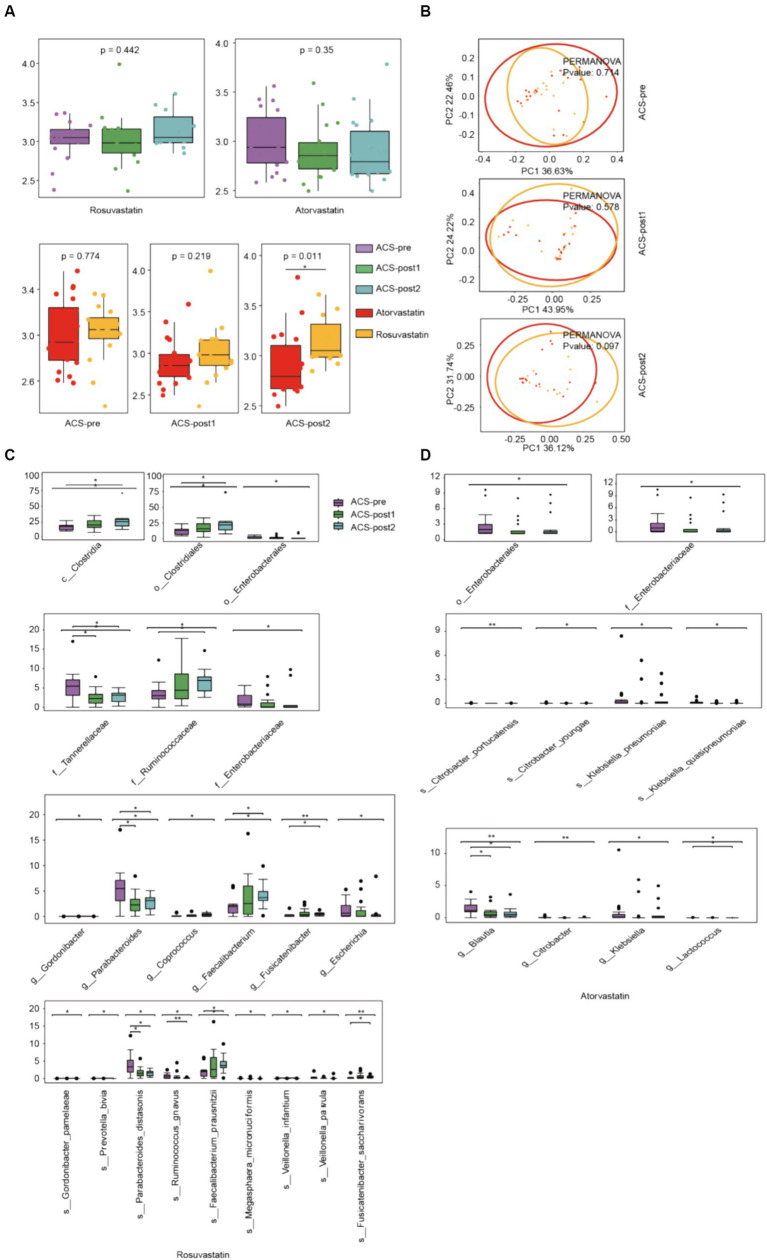
Alterations in gut microbiota affected by drugs. **(A)** α-diversity (Shannon index) of ACS patients treated by different drugs at three stages of therapy. **(B)** Principal Coordinates Analysis (PCoA) of patients used different medications at three stages of therapy (Bray–Curtis distance). **(C)** Microbiota that changed significantly after treatment with rosuvastatin. **(D)** Microbiota that changed significantly after treatment with atorvastatin (*adjust *p* < 0.05, **adjust *p* < 0.01, and ***adjust *p* < 0.001, Kruskal–Wallis test, Benjamini–Hochberg FDR).

**Figure 4:**
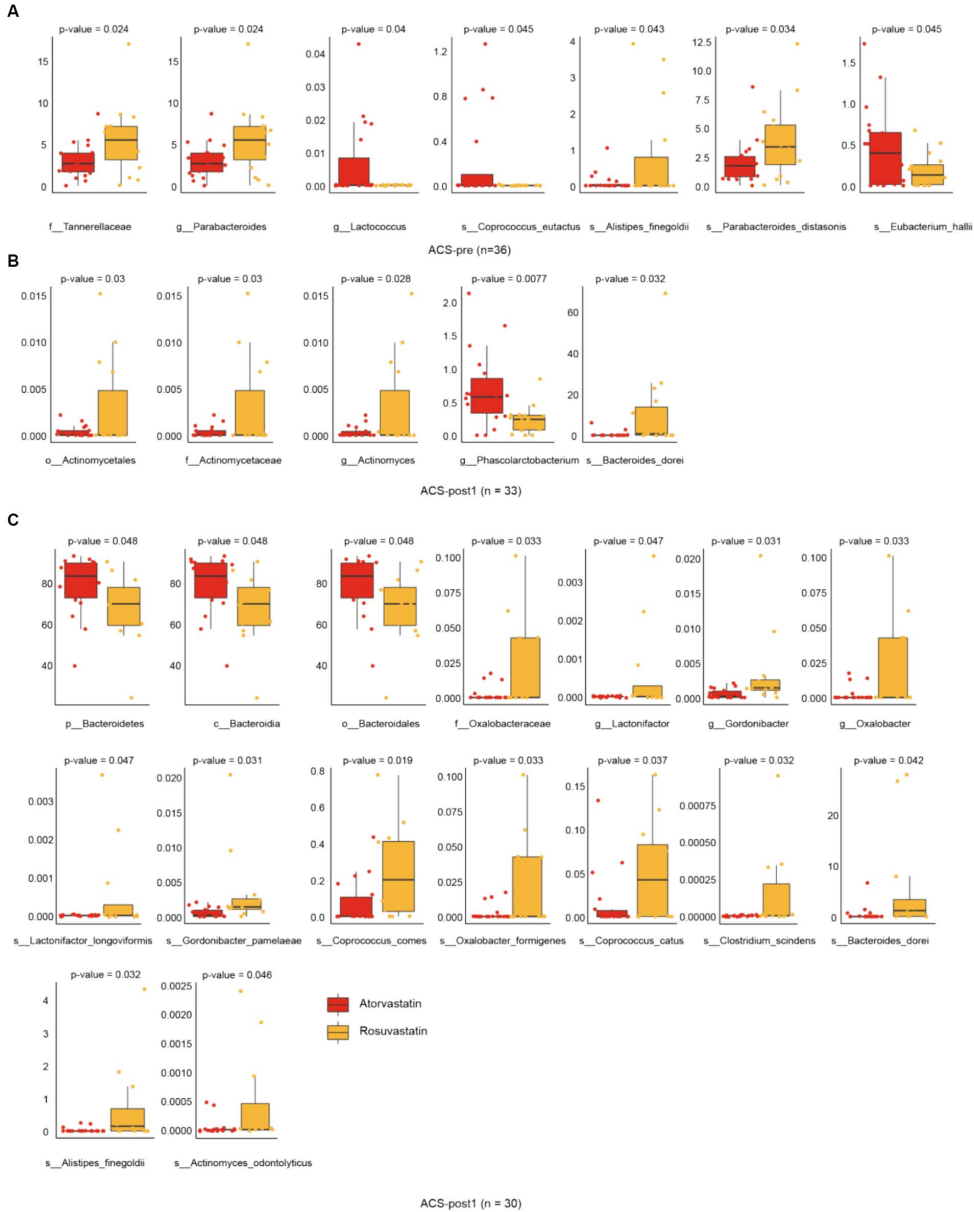
Alterations in gut microbiota affected by drugs. **(A)** Differential species in groups atorvastatin and rosuvastatin before treatment. **(B)** Differential species in groups atorvastatin and rosuvastatin one month after treatment. **(C)** Differential species in groups atorvastatin and rosuvastatin 2 month after treatment (*t*-test, Benjamini–Hochberg FDR).

With different stages of treatment, there were more significant differences in gut microbial alpha (Shannon) diversity between patients using two different drugs, rosuvastatin (R) and atorvastatin (A) (Figure 3A). Principal Coordinates Analysis (PCoA) by Bray Curtis distance of patients’ intestinal microorganisms also showed the same trend (Figure 3B). After treatment with rosuvastatin, there were significant changes in the relative abundances of six genera and nine species between ACS-pre and ACS-post (Figure 3C). These changes included increases in the abundances of *Gordonibacter*, *Coprococcus*, *Faecalibacterium*, and *Fusicatenibacter*, and decreases in the abundance of *Escherichia* and *Parabacteroides*. The relative abundances of specific species also showed significant alterations: *Gordonibacter pamelaeae*, *Prevotella bivia*, *Parabacteroides distasonis*, *Ruminococcus gnavus*, *Faecalibacterium prausnitzii*, *Megasphaera micronuciformis*, *Veillonella infantium*, *Veillonella parvula*, and *Fusicatenibacter saccharivorans*. After treatment with atorvastatin, there were significant changes in the relative abundances of four genera and four species (Figure 4D). Furthermore, we also analyzed the differences between different microbiota at three treatment stages (Figure 4). Before treatment, there were 7 significantly different bacteria between groups A and R: *Tannerellaceae* (family); *Parabacteroides* (genus); *Alistipes finegoldii*, *Parabacteroides distasonis*, *Eubacterium hallii*, *Coprococcus eutactus* (species) (Figure 4A). After 1 month of treatment, there were 5 significantly different species between the two groups, namely: *Actinomycetales* (order); *Actinomycetaceae* (family); *Actinomyces*, *Phascolarctobacterium* (genus); *Bacteroides dorei* (species) (Figure 4B). After 2 months of treatment, there were 16 significantly different species between the two groups: *Bacteroidetes* (phylum); *Bacteroidia* (class); *Bacteroidales* (order); *Oxalobacteraceae* (family); *Gordonibacter*, *Lactonifactor*, *Oxalobacter* (genus); *Actinomyces odontolyticus*, *Gordonibacter pamelaeae*, *Bacteroides dorei*, *Alistipes finegoldii*, *Lactonifactor longoviformis*, *Coprococcus catus*, *Coprococcus comes*, *Clostridium scindens*, *Oxalobacter formigenes* (species) (Figure 4C).

## Methods

### ACS patient recruitment and specimen collection

Faecal samples from 38 ACS patients and family members with an equal number were collected at the Zhuhai People’s Hospital (Zhuhai Clinical Medical College of Jinan University) between January and June 2023. All patients were Han Chinese with no known consanguinity, newly diagnosed with ACS. All the family members of ACS patients used as healthy controls were free of clinically obvious ACS symptoms. Ethical clearance was obtained from the IRB of Zhuhai People’s Hospital (Zhuhai Clinical Medical College of Jinan University) (approved no. 24, 2020). Informed consent was obtained from the patients. On the morning of hospital admission, one month after percutaneous coronary intervention and during the second month post-treatment, sterile swabs were utilized to collect mid-section fresh faecal samples from patients. All samples were rapidly frozen on dry ice within 30 min and preserved in −80°C freezers before DNA extraction. Blood samples of patients were collected in the morning after an overnight fast. Plasma was collected by centrifugation and stored in −80°C freezers.

### Pathological plasma biochemical analysis

Biochemical parameters, renal function parameters, lipids, coagulation factors, and blood cell counts were measured by AU2700 fully automatic blood biochemical analyzer. A total of 9 indicators were analyzed, including triglycerides (TG), cholesterol (Chol), high-density lipoprotein (HDL), low-density lipoprotein (LDL), albumin (ALB), Creatinine (Cr), cardiac troponin T (cTnT), B-type natriuretic peptides (BNP), hemoglobin A1c (HbA1c). Ejection fraction (EF) was measured by echocardiography.

### Genomics DNA extraction

The microbial community DNA was extracted using MagPure Stool DNA KF kit B (Magen, China) following the manufacturer’s instructions. DNA was quantified with a Qubit Fluorometer by using a Qubit dsDNA BR Assay kit (Invitrogen, United States), and the quality was checked by running an aliquot on 1% agarose gel. The quality of the DNA from all samples was quality controlled.

### Library construction and sequencing

DNA was randomly fragmented by Covaris. The fragmented DNA was selected by Magnetic beads to an average size of 200–400 bp. The selected fragments were processed through end-repair, 3′ adenylated, adapters-ligation, and PCR Amplifying, and the products were purified using magnetic beads. The double-stranded PCR products were heat-denatured and circularized by the splint oligo sequence. The single-strand circle DNA (ssCir DNA) was formatted as the final library and qualified by QC. The qualified libraries were sequenced on the BGISEQ-500 platform (BGI-Shenzhen, China).

### Metagenomic data analysis

Taxonomic and functional profiling of microbial communities was generated with tools from the biobakery meta-omics analysis environment^1^ (McIver et al., 2018). Reads mapping to the human genome were first subjected to initial quality control via KneadData v0.10.0. Taxonomic profiling of metagenome detection was conducted using MetaPhlAn3 v3.0.14 with the default parameters. Microbiome relative abundance was assessed using Metaphlan3. The v3.0.14 CHOCOPhlAn database was used to generate the microbial relative abundances for each sample. The relative abundance profiles of gene presence and abundance were determined by mapping reads to the UniRef90 protein reference database (Suzek et al., 2015). Functional profiling was performed by HUMAnN3 v3.0.1 with the default parameters.

### Statistical methods and visualization

Diversity was analyzed and visualized with GutMeta online tools^2^. Differential pathways were identified using humann2_associate in HUMAnN2 (Franzosa et al., 2018), and the species composition of differential pathways was plotted using humann2_barplot. Differential species were calculated using the LEFSe method (Segata et al., 2011). The potential biomarkers of functional pathways were assessed using STAMP (Parks et al., 2014) v2.1.3. PermANOVA was performed using adonis2, and the mantel test was computed with 9,999 permutations, both using R package vegan (Oksanen et al., 2019). Spearman correlations were calculated using R. The statistical test was performed using the *t*-test (for two groups) and Kruskal–Wallis test (for three groups and more). Benjamini–Hochberg correction for multiple comparisons was applied (Wang, 2022). Adjust *p* < 0.05 was considered statistically significant.

## Discussion

The study investigated the changes in gut microbiota composition in patients with ACS before and after treatment. The study found 16 significant species changes driven by ACS and identified 39 differential bacteria before and after treatment. The gut microbiota of patients returned to a relatively stable state 1 month after treatment. The relative abundances of seven pathways related to the biosynthesis of vitamin K2 and its homolog were reduced. Additionally, two pathways related to sugar degradation were more abundant in patients before treatment. The research also found that the gut microbiota of ACS patients treated with atorvastatin and rosuvastatin exhibit unique states. Following treatment with rosuvastatin, there was an increase in the richness of gut microbiota among patients, while patients treated with atorvastatin showed contrasting outcomes. These findings provide insight into specific bacteria and metabolic pathways that may be associated with cardiovascular disease risk and offer opportunities for treatment.

Our findings revealed no significant effect of ACS and its treatment on microbial diversity, which is consistent with some previous studies but contradicts others (Liu et al., 2019; Yoshida et al., 2019; Liu et al., 2020; Toya et al., 2020; Ma et al., 2021). Regarding the factors responsible for the differences in gut microbes, we found that inter-individual differences accounted for a relatively large proportion, although other physiological and pathological factors only explained a small proportion of the variance, which aligned with previous findings. Among these factors, cTnT is an essential biomarker for diagnosing ACS, particularly myocardial infarction, and can also be used to predict prognosis and mortality (Árnadóttir et al., 2018; Desai et al., 2020). BNP is frequently used to assess the short- and intermediate-term prognosis in ACS patients (Bassan et al., 2016). Therefore, we hypothesized that cTnT and BNP may also be involved in the development of ACS by affecting the percentages of metabolites and microbial species.

Alterations in the gut microbiome (dysbiosis) have been shown to cause chronic inflammation^3^ (Buttó and Haller, 2016; Li et al., 2019b; Moludi et al., 2021)—a key factor in ACS. It has been suggested that *Firmicutes* and *Bacteroidetes* play a role in inflammation due to their short-chain fatty acids (SCFAs) production (Tang and Hazen, 2014; Kim, 2018). *Firmicutes* mainly produce butyrate, while *Bacteroidetes* produce acetate and propionate (Magne et al., 2020). Butyrate favours cardiovascular disease-related disorders, as it induces AMPK activation and GLUT4 expression in adipose tissue, suppresses atherosclerotic plaque formation, and reduces reactive oxygen species (Gao et al., 2019; Mamic et al., 2021). However, acetate is considered a risk factor for obesity, as it induces lipid accumulation in the liver and stimulates appetite (Lecomte et al., 2015; Magne et al., 2020). Some *Bacteroidetes* spp., such as *B. thetaiotaomicron*, can contribute to diet-induced obesity and hypertension (Tan et al., 2019; Calderón-Pérez et al., 2020; Cho et al., 2022). Therefore, the *Firmicutes*/*Bacteroidetes* ratio was higher in healthy individuals than in patients with coronary heart disease, which could explain our result. Our study found that the *Firmicutes*/*Bacteroidetes* ratio was lower in ACS patients compared to healthy subjects, but this ratio increased after therapy. Moreover, *Parabacteroides*, *Escherichia*, and *Blautia* were significantly abundant in patients before treatment in our study. A higher abundance of *Blautia* has been associated with an increased risk of developing coronary artery disease in the Chinese population and may contribute to diseases through the production of pro-inflammatory metabolites and depletion of beneficial gut bacteria (Liu et al., 2019). In addition, *Blautia* can produce butyric acid, which is involved in forming short-chain fatty acids (SCFAs) (Liu et al., 2019). We also found that *A. colihominis*, *C. evryensis*, and *F. fastidiosum* significantly increased in ACS patients. *F. fastidiosum* is a typical oral bacterium which is often present in patients with periodontal disease (Deng et al., 2017; Krishnan et al., 2017). Periodontal disease is a risk factor for cardiovascular disease (Khalighinejad et al., 2016; Deng et al., 2017), generally manifested by increased inflammation and potential changes in hypercoagulability and insulin resistance (Mathews et al., 2016). The increase in the abundance of pro-inflammatory bacteria may be associated with ACS disease.

Also, gut bacteria produce several metabolites that may influence heart health. We found that the abundance of *G. pamelaeae*, *E. eligens*, *B. hansenii*, and *R. ilealis* decreased. The reduction of these species and metabolites may lead to elevated substances associated with cardiovascular disease in humans, such as cholesterol, visceral fat area, visceral fat mass, and plasma high-density lipoprotein. *G. pamelaeae* can produce urolithin (Selma et al., 2014), which can be anti-atherosclerotic (Stromsnes et al., 2020) and prevent cardiometabolic risk (Furlanetto et al., 2012; Giménez-Bastida et al., 2012; Selma et al., 2018). *E. eligens* produces butyric acid, which inhibits obesity and prevents coronary heart disease (Jie et al., 2017). Other studies also found that ACS patients had fewer *E. eligens* in the gut (Liu et al., 2020; Nakai et al., 2021). There is a decrease in the abundance of beneficial bacterial strains in ACS patients.

Otherwise, we have identified certain intestinal flora metabolic pathways that can potentially impact the development and treatment of ACS. Sugar metabolism, particularly glucose metabolism, plays a significant role in the development and progression of ACS (Mao et al., 2019). Our study found that pathways related to sugar degradation were less abundant after treatment, while pathways related to sugar molecule biosynthesis were more abundant. This suggests that the glucose metabolism disorder in the patient’s body improved after treatment. Our study revealed the importance of vitamin K2, specifically menaquinone-7, in regulating calcium deposition in the arterial wall and preventing arterial calcification, a key risk factor for atherosclerosis and subsequent acute coronary events (Mandatori et al., 2021). Higher dietary intake or supplementation of vitamin K2 has been associated with a lower risk of cardiovascular disease, including ACS (Schurgers and Vermeer, 2000; Beulens et al., 2009; Vissers et al., 2013; Knapen et al., 2015). Our study revealed that seven pathways related to the biosynthesis of vitamin K2 and its homolog were reduced after treatment. This may indicate that the patient’s symptoms related to atherosclerosis were relieved, and there was no need to produce more vitamin K2.

Our findings suggest that rosuvastatin and atorvastatin have distinct effects on the gut microbiota composition in acute coronary syndrome patients. After two months of treatment, the intestinal microbial diversity of ACS patients taking rosuvastatin showed an increasing trend, while the intestinal microbial diversity of ACS patients taking atorvastatin showed the opposite decreasing trend. Rosuvastatin was associated with increased gut microbiota richness, consistent with previous research (Liu et al., 2018; Kummen et al., 2020). It is speculated that atorvastatin treatment of hypercholesterolemia can selectively restore the relative abundance of several dominant and functionally important taxa that are disrupted after illness, but further studies are needed to verify this (Khan et al., 2018). Specifically, rosuvastatin promoted the growth of anti-inflammatory bacteria, such as *F. prausnitzii* (Sokol et al., 2008; Khan et al., 2018; Saber et al., 2021), while reducing the abundance of pro-inflammatory bacteria, including *Escherichia* (Nolan et al., 2017), *R. gnavus* (Henke et al., 2021), *P. bivia* (Strömbeck et al., 2007; Hou et al., 2022), and *V. parvula* (Zhan et al., 2022). *F. prausnitzii* is known for its anti-inflammatory effects, including the inhibition of NF-κB activation and IL-8 production (Breyner et al., 2017; Lopez-Siles et al., 2017; Lenoir et al., 2020). It also produces butyrate, which has anti-inflammatory properties. Conversely, certain strains of *R. gnavus* have been associated with inflammatory bowel disease and other inflammatory conditions (Hall et al., 2017). *Veillonella* has been found to induce inflammation in clinical conditions (Zhan et al., 2022; Zeng et al., 2023). Atorvastatin decreased the levels of pro-inflammatory bacteria such as *Enterobacteriaceae* (Zeng et al., 2017; Baldelli et al., 2021), as well as anti-inflammatory bacteria like *Blautia* and *Lactococcus* (Ma et al., 2023). *Lactococcus*, particularly *Lactococcus lactis*, has demonstrated potential as a probiotic strain with anti-inflammatory properties, particularly in treating colitis and mucositis (Luerce et al., 2014; Liu et al., 2019). It has been shown to have immunomodulatory activity, enhancing Th1-type immune responses, which can contribute to anti-inflammatory effects. Additionally, atorvastatin was associated with increased abundance of the *Bacteroides* genus. Studies have shown that atorvastatin treatment increased the relative abundance of *Bacteroides* genera in high-fat diet-induced hypercholesterolemic rats (Khan et al., 2018). Rosuvastatin and atorvastatin both affect the gut microbiota through variability in FXR receptor signalling. Rosuvastatin alters the host gene expression of bile acid metabolism pathways, while atorvastatin leads to decreased secondary bile acids (Tuteja and Ferguson, 2019). These results contribute to our understanding of the potential microbiota-related mechanisms underlying the therapeutic effects of these statins.

This study has several limitations expected to be improved in future research. Firstly, we included a relatively small cohort primarily due to losing many patients during follow-up. Inter-individual variation accounted for 69.6% of the variance in our study’s relative abundance of species’ levels, a common issue in metagenomic research. To address the sample size limitation, we employed stringent inclusion criteria to reduce individual differences. We utilized metagenomic sequencing to maximize the information extracted from each sample. Secondly, the cross-sectional nature of this study involved sampling patients from only one hospital in Zhuhai, which may not fully capture the diversity of the broader population, thus constraining the generalizability of the findings. Thirdly, the intricate interplay among medication use, gut microbiota, and confounding factors presents several limitations in our study. Medication administration signifies alterations in the host’s health status, which may be accompanied by changes in lifestyle factors (such as smoking, alcohol consumption, and physical activity) known to impact gut microbiota composition. While we controlled for dietary patterns (within the same regional population) and age (within a specific age range), we could not regulate the influence of comorbidities (such as diabetes or obesity), medications (particularly antibiotics, as well as proton pump inhibitors, nonsteroidal anti-inflammatory drugs, and metformin), and genetics (inter-individual genetic variability). Fourthly, this study solely relies on empirical analysis using genome sequencing data and lacks animal experiments to delve deeper into the mechanisms underpinning these observations. Considering the limitations above, we intend to gather a larger sample size to monitor taxonomic and functional changes in gut microbiota to validate the existing findings. Furthermore, we aim to incorporate animal experiments to validate the underlying mechanisms.

In summary, we identified risk biomarkers between ACS patients and healthy individuals and detected significant alterations in the gut microbiota of ACS patients before and after treatment. We also analyzed the differences in gut microbiomes in patients after using two different statins. These may mediate the development and treatment of ACS by translating into metabolisms. Further research utilizing advanced multi-omic detection and joint analysis techniques in prospective studies is necessary to better understand the role of gut microbiota in the pathogenesis of coronary heart disease and identify potential therapeutic targets.

## Data availability statement

The datasets presented in this study can be found in online repositories. The names of the repository/repositories and accession number(s) can be found in the article/Supplementary material.

## Ethics statement

The studies involving humans were approved by Ethical clearance was obtained from the IRB of Zhuhai People’s Hospital (Zhuhai Clinical Medical College of Jinan University) (approve no. 24, 2020). The studies were conducted in accordance with the local legislation and institutional requirements. The participants provided their written informed consent to participate in this study. Written informed consent was obtained from the individual(s) for the publication of any potentially identifiable images or data included in this article.

## Author contributions

YG: Writing – original draft, Investigation, Resources, Funding acquisition. SZ: Formal analysis, Writing – original draft, Visualization, Writing – review & editing. JL: Formal analysis, Writing – original draft. WZ: Formal analysis, Writing – original draft. ZG: Formal analysis, Writing – original draft. YL: Investigation, Writing – original draft. XJ: Project administration, Writing – review & editing. JuL: Investigation, Resources, Writing – original draft. JiL: Investigation, Resources, Writing – original draft. XC: Writing – review & editing, Investigation, Funding acquisition. ZiZ: Conceptualization, Methodology, Writing – review & editing. ZhZ: Conceptualization, Project administration, Funding acquisition, Methodology, Resources, Writing – review & editing.

## Footnotes

^1^http://huttenhower.sph.harvard.edu/biobakery

^2^https://gutmeta.deepomics.org/

^3^参考文献: https://www.ncbi.nlm.nih.gov/pmc/articles/PMC8170788/
